# Supplementary material for: Adaptive resampling for improved machine learning in imbalanced single-cell datasets
Source: bioRxiv. 2025 Nov 5:2025.11.04.686583. Preprint. [Version 1] doi: 10.1101/2025.11.04.686583 (PMC12637690; doi:10.1101/2025.11.04.686583)
Supplement: 1 [file NIHPP2025.11.04.686583v1-supplement-1.pdf]

bioRxiv preprint doi: https://doi.org/10.1101/2025.11.04.686583; this version posted November 5, 2025. The copyright holder for this preprint (which was not certified by peer review) is the author/funder, who has granted bioRxiv a license to display the preprint in perpetuity. It is made available under aCC-BY 4.0 International license.

744

Supplementary Figures and Tables

Table S1. Mean and standard deviation of Heart, Kidney, and Neuron cell counts included during scVI model training for five different seeds across different scenarios where 0% (0 cells), 0.001% (1 cell), 0.01% (10 cells), 0.1% (100 cells), 1% (1k cells), 10% (10k cells), and 50% (50k cells) of the total 100k training samples were randomly selected from the atlas population and combined with the base blood cell population.

# Atlas Cells	0	1	10	100	1000	10000	50000
Heart	0.0 ± 0.0	0.0 ± 0.0	0.4 ± 0.5	2.6 ± 1.5	19.8 ± 1.5	179.4 ± 7.8	928.2 ± 31.3
Kidney	0.0 ± 0.0	0.0 ± 0.0	0.2 ± 0.4	0.8 ± 0.8	12.2 ± 2.6	122.2 ± 10.4	593.8 ± 9.9
Neuron	0.0 ± 0.0	0.0 ± 0.0	1.8 ± 0.4	23.4 ± 3.1	224.6 ± 7.1	2221.2 ± 20.7	11256.8 ± 34.2

Table S2. The p-values for two-sided t-tests on evaluation metrics for the AR and standard scVI models tested on the Heart dataset. Bold indicates statistical significance as determined by $P < 0.05$. Results are based on five replicates.

# Atlas Cells	0	1	10	100	1000	10000	50000
Accuracy	**1.83e-03**	**1.60e-04**	**1.54e-02**	**3.96e-03**	**2.73e-07**	**7.70e-07**	**4.30e-04**
Macro F1 Score	**2.66e-02**	**2.62e-03**	**1.12e-02**	**1.18e-03**	**2.55e-06**	**1.23e-04**	**3.14e-03**
Micro F1 Score	**1.83e-03**	**1.60e-04**	**1.54e-02**	**3.96e-03**	**2.73e-07**	**7.70e-07**	**4.30e-04**
Precision	**1.25e-02**	**9.37e-03**	**3.14e-03**	**6.78e-03**	**1.28e-05**	**1.27e-04**	**1.06e-02**
Recall	**4.87e-02**	**1.97e-03**	**2.33e-02**	**1.28e-03**	**3.67e-06**	**7.86e-05**	**6.26e-03**
Correlation (R^2)	**2.08e-05**	**3.97e-05**	**4.28e-05**	**3.94e-09**	**2.01e-07**	**2.18e-11**	**2.45e-06**

bioRxiv preprint doi: https://doi.org/10.1101/2025.11.04.686583; this version posted November 5, 2025. The copyright holder for this preprint (which was not certified by peer review) is the author/funder, who has granted bioRxiv a license to display the preprint in perpetuity. It is made available under aCC-BY 4.0 International license.

Table S3. The p-values for two-sided t-tests on evaluation metrics for the AR and standard scVI models tested on the Kidney dataset. Bold indicates statistical significance as determined by $P < 0.05$. Results are based on five replicates.

# Atlas Cells	0	1	10	100	1000	10000	50000
Accuracy	**4.10e-02**	**3.72e-02**	9.38e-02	**6.75e-03**	**1.44e-04**	**1.56e-05**	**2.29e-04**
Macro F1 Score	**2.11e-02**	**9.16e-03**	**1.18e-02**	**8.74e-03**	**2.40e-05**	**1.35e-05**	**6.56e-04**
Micro F1 Score	**4.10e-02**	**3.72e-02**	9.38e-02	**6.75e-03**	**1.44e-04**	**1.56e-05**	**2.29e-04**
Precision	**1.14e-02**	**2.37e-05**	**2.32e-05**	**1.91e-03**	**5.55e-05**	**3.69e-06**	**2.67e-03**
Recall	5.07e-02	**2.04e-02**	7.82e-02	**1.57e-02**	**2.21e-05**	**1.66e-05**	**2.25e-04**
Correlation (R^2)	**4.81e-07**	**8.99e-07**	**4.53e-05**	**1.96e-10**	**2.64e-07**	**6.01e-07**	**2.89e-03**

Table S4. The p-values for two-sided t-tests on evaluation metrics for the AR and standard scVI models tested on the Neuron dataset. Bold indicates statistical significance as determined by $P < 0.05$. Results are based on five replicates.

# Atlas Cells	0	1	10	100	1000	10000	50000
Accuracy	**8.77e-03**	**1.80e-02**	9.06e-02	**1.33e-04**	**1.91e-07**	**9.52e-07**	1.62e-01
Macro F1 Score	**5.91e-03**	**2.33e-03**	1.72e-01	**3.48e-04**	**9.48e-07**	**3.99e-06**	5.76e-01
Micro F1 Score	**8.77e-03**	**1.80e-02**	9.06e-02	**1.33e-04**	**1.91e-07**	**9.52e-07**	1.62e-01
Precision	7.97e-02	**3.46e-02**	1.21e-01	**1.01e-04**	**3.11e-05**	**6.11e-05**	8.89e-01
Recall	**6.00e-03**	**2.78e-03**	1.87e-01	**2.24e-04**	**5.55e-07**	**1.51e-06**	5.25e-01
Correlation (R^2)	**7.35e-05**	**6.87e-05**	**6.18e-05**	**1.51e-10**	**1.19e-05**	**4.52e-06**	5.29e-01

bioRxiv preprint doi: https://doi.org/10.1101/2025.11.04.686583; this version posted November 5, 2025. The copyright holder for this preprint (which was not certified by peer review) is the author/funder, who has granted bioRxiv a license to display the preprint in perpetuity. It is made available under aCC-BY 4.0 International license.

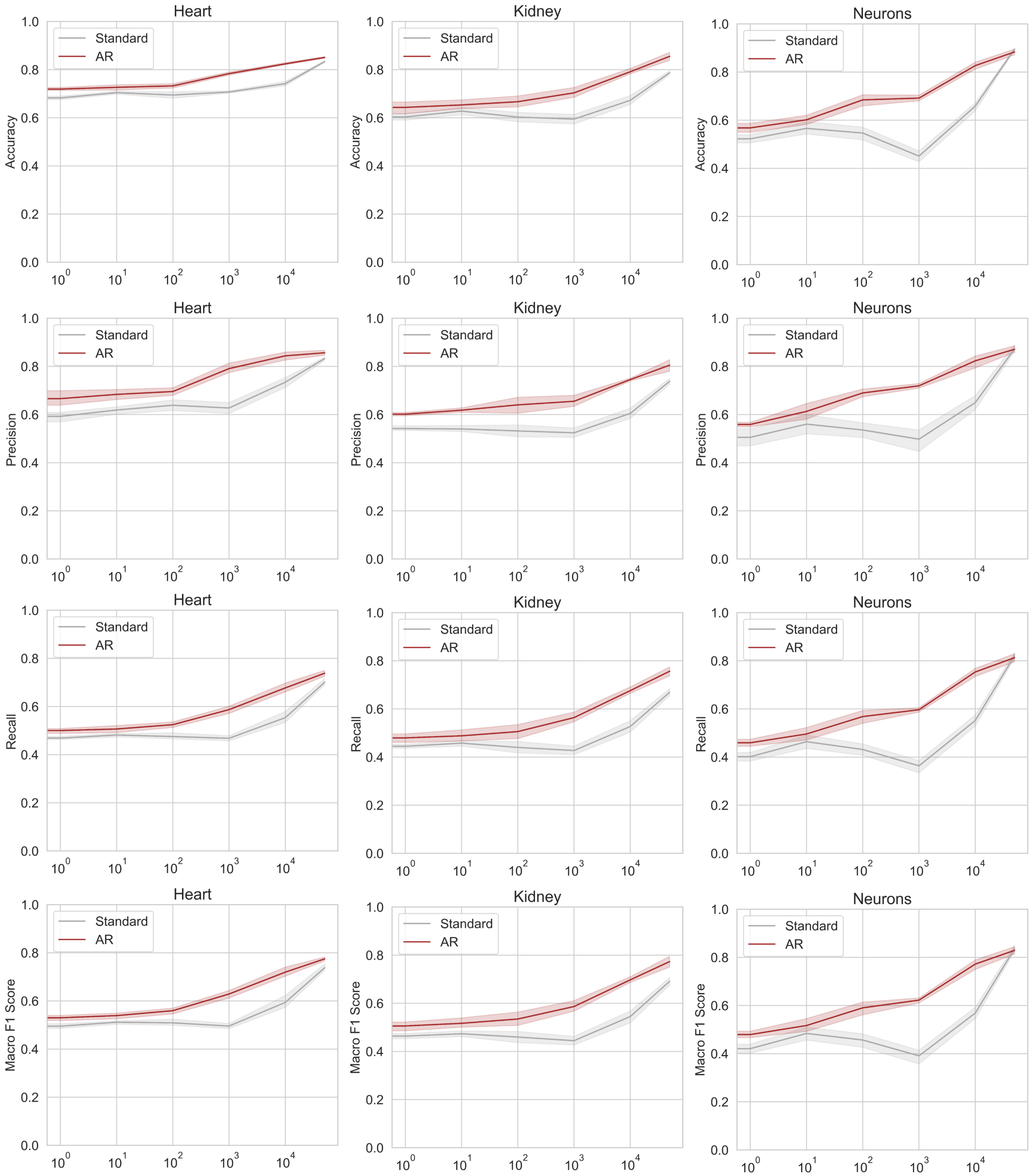

Figure S1. Cell type classification performance on Heart, Kidney, and Neuron evaluation datasets. Classification is done using the model embeddings from scVI models trained either in the standard way (grey) or with AR (red). Performance is measured by the accuracy, precision, recall, and macro F1 scores. The x-axis represents the number of atlas cells included in the blood-based training dataset, with all training sets having a total of 100k cells. Results are based on five different random seeds, with error bars representing the 95% confidence interval across replicates.

bioRxiv preprint doi: https://doi.org/10.1101/2025.11.04.686583; this version posted November 5, 2025. The copyright holder for this preprint (which was not certified by peer review) is the author/funder, who has granted bioRxiv a license to display the preprint in perpetuity. It is made available under aCC-BY 4.0 International license.

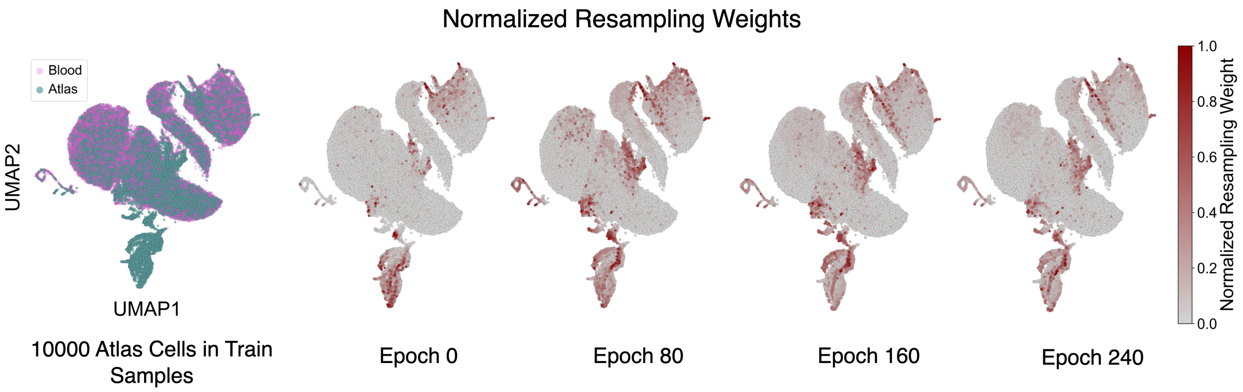

Figure S2. Underrepresented atlas cells are assigned higher resampling weights compared to blood cells in the training corpus. Uniform manifold approximation and projection (UMAP) visualizations of training samples for one random seed of the 90,000 blood cell + 10,000 atlas cell dataset, annotated by their cell group (left; blood in purple and atlas in green) and by normalized resampling weights over the course of AR model training (right).

bioRxiv preprint doi: https://doi.org/10.1101/2025.11.04.686583; this version posted November 5, 2025. The copyright holder for this preprint (which was not certified by peer review) is the author/funder, who has granted bioRxiv a license to display the preprint in perpetuity. It is made available under aCC-BY 4.0 International license.

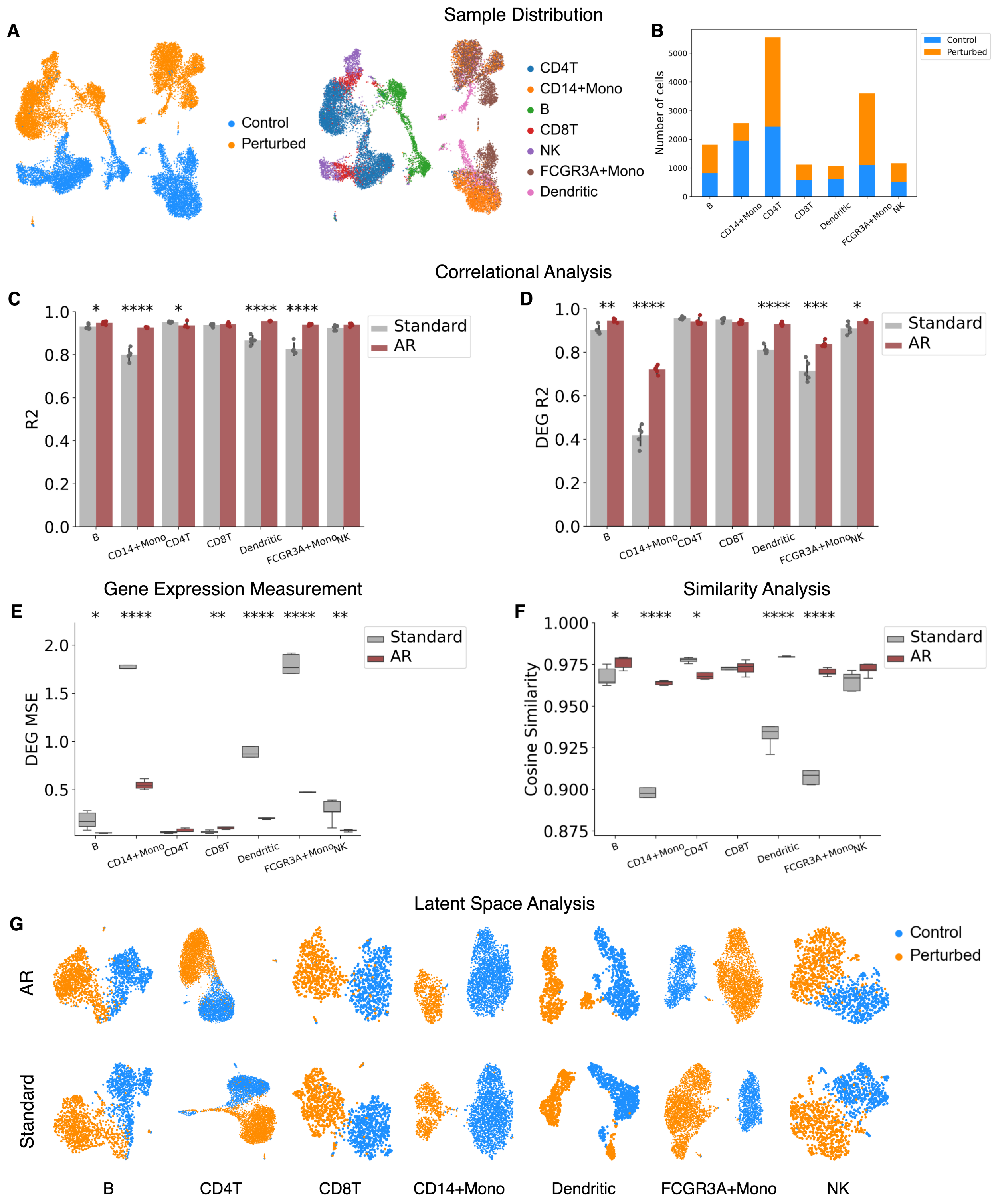

Figure S3. Adaptive resampling enables robust prediction of cellular response to IFN-β stimulation in human immune cells. (A) UMAP visualization of sample distributions in the PBMC dataset, annotated by condition (control versus perturbed, left) and by cell groups (right). **(B)** Cell counts per condition and cell group. **(C-D)** Square of the Pearson correlation coefficient between real and predicted perturbed gene expression using AR versus standard models for each held-out test cell group across (C) all genes and (D) only differentially expressed genes. Results are based on five different random seeds, with error bars representing the standard deviations across replicates. **(E)** Mean sqaured error (MSE) computed between ground-truth and predicted perturbed gene expression profiles across differentially expressed genes for AR versus standard models. **(F)** Distributions of cosine similarity scores for predicted expression profiles across all genes, relative to ground-truth expression profiles. **(G)** UMAP visualizations of the latent space projections for test samples, annotated by condition, comparing AR and standard learned latent embeddings. Two-sided t-tests were performed in all experiments to compare the means of metrics, with $^{*}P < 0.05$, $^{**}P < 0.01$, $^{***}P < 0.001$, and $^{****}P < 0.0001$.

bioRxiv preprint doi: https://doi.org/10.1101/2025.11.04.686583; this version posted November 5, 2025. The copyright holder for this preprint (which was not certified by peer review) is the author/funder, who has granted bioRxiv a license to display the preprint in perpetuity. It is made available under aCC-BY 4.0 International license.

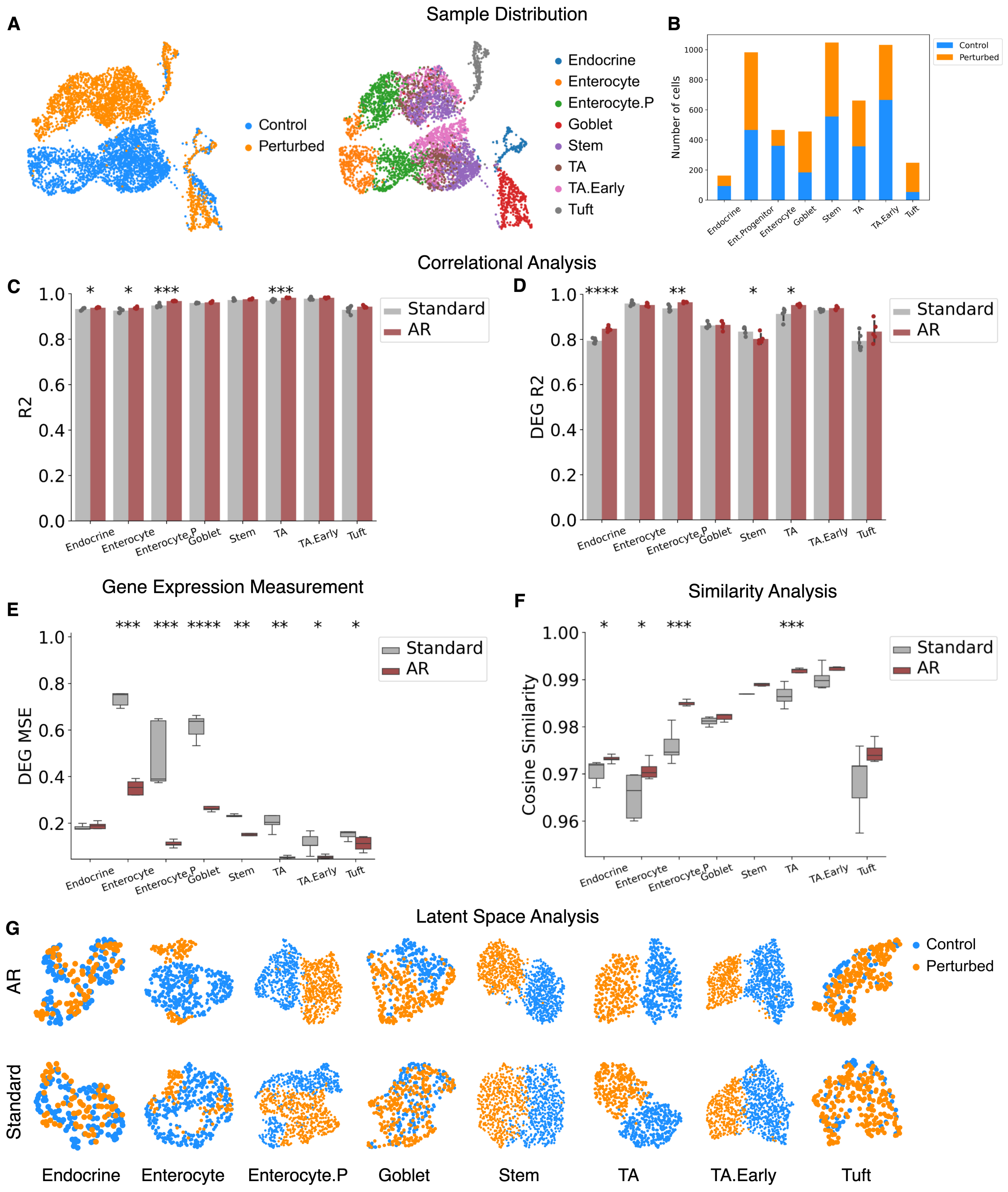

Figure S4. Adaptive resampling enables robust prediction of cellular response to *H. poly* parasitic infection in the murine intestinal epithelium. **(A)** UMAP visualization of sample distributions in the H. poly dataset, annotated by condition (control versus perturbed, left) and by cell groups (right). **(B)** Cell counts per condition and cell group. **(C-D)** Square of the Pearson correlation coefficient between real and predicted perturbed gene expression using AR versus standard models for each held-out test cell group across (C) all genes and (D) only differentially expressed genes. Results are based on five different random seeds, with error bars representing the standard deviations across replicates. **(E)** Mean sqaured error (MSE) computed between ground-truth and predicted perturbed gene expression profiles across differentially expressed genes for AR versus standard models. **(F)** Distributions of cosine similarity scores for predicted expression profiles across all genes, relative to ground-truth expression profiles. **(G)** UMAP visualizations of the latent space projections for test samples, annotated by condition, comparing AR and standard learned latent embeddings. Two-sided t-tests were performed in all experiments to compare the means of metrics, with $^{*}P < 0.05$, $^{**}P < 0.01$, $^{***}P < 0.001$, and $^{****}P < 0.0001$.

bioRxiv preprint doi: https://doi.org/10.1101/2025.11.04.686583; this version posted November 5, 2025. The copyright holder for this preprint (which was not certified by peer review) is the author/funder, who has granted bioRxiv a license to display the preprint in perpetuity. It is made available under aCC-BY 4.0 International license.

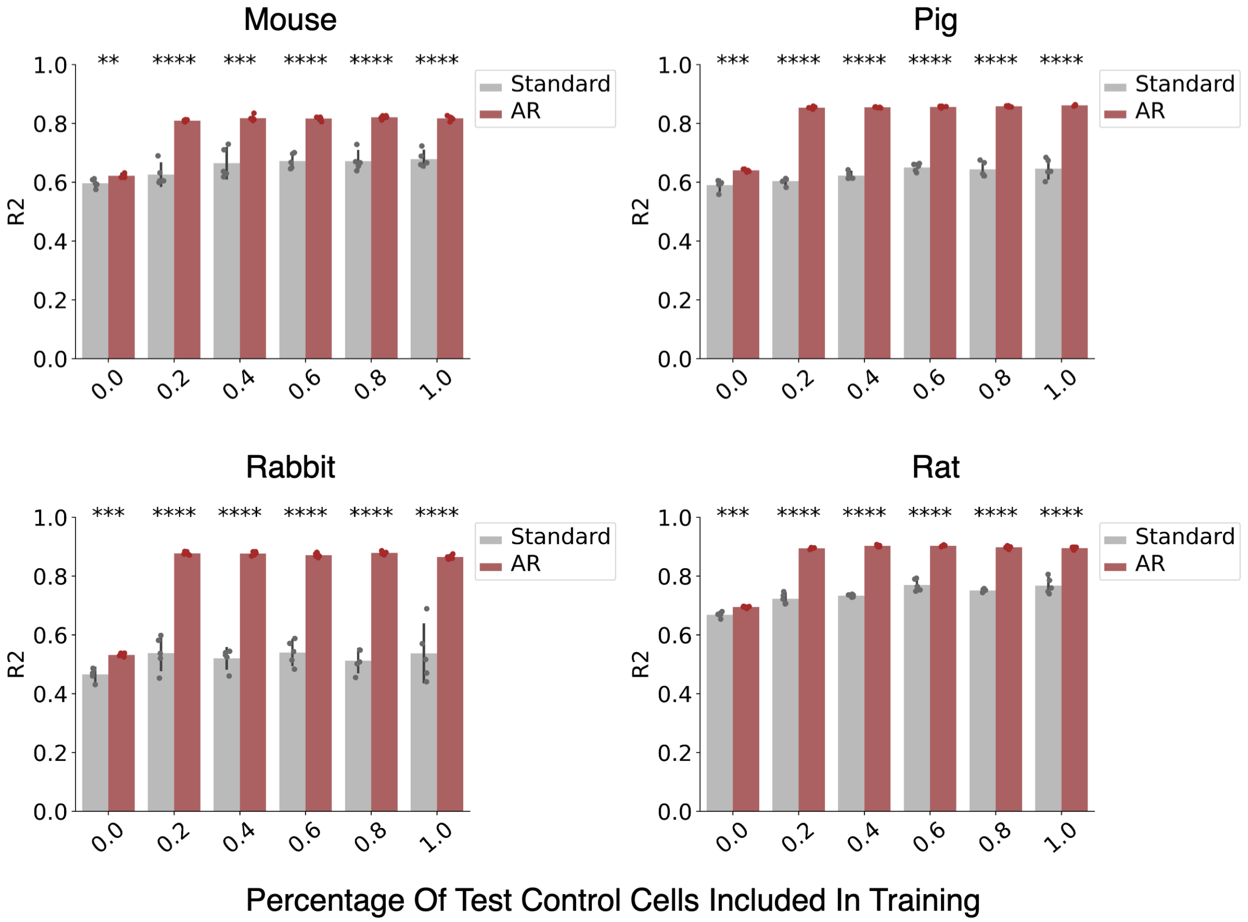

Figure S5. Adaptive resampling enables generalizable perturbation response prediction on species that are underrepresented or completely unseen. Square of the Pearson correlation coefficient (R^2) between ground-truth and predicted perturbed gene expression in rare sample generalization scenarios on the Species dataset for AR versus standard models. Individual plots show results where we trained models while incrementally introducing varying proportions of control cells from the held-out group into the training corpus—specifically, 0%, 20%, 40%, 60%, 80%, and 100%. Results are based on five different random seeds, with error bars representing the standard deviations across replicates. Two-sided t-tests were performed in all experiments to compare the means of metrics, with $^{*}P < 0.05$, $^{**}P < 0.01$, $^{***}P < 0.001$, and $^{****}P < 0.0001$.

bioRxiv preprint doi: https://doi.org/10.1101/2025.11.04.686583; this version posted November 5, 2025. The copyright holder for this preprint (which was not certified by peer review) is the author/funder, who has granted bioRxiv a license to display the preprint in perpetuity. It is made available under aCC-BY 4.0 International license.

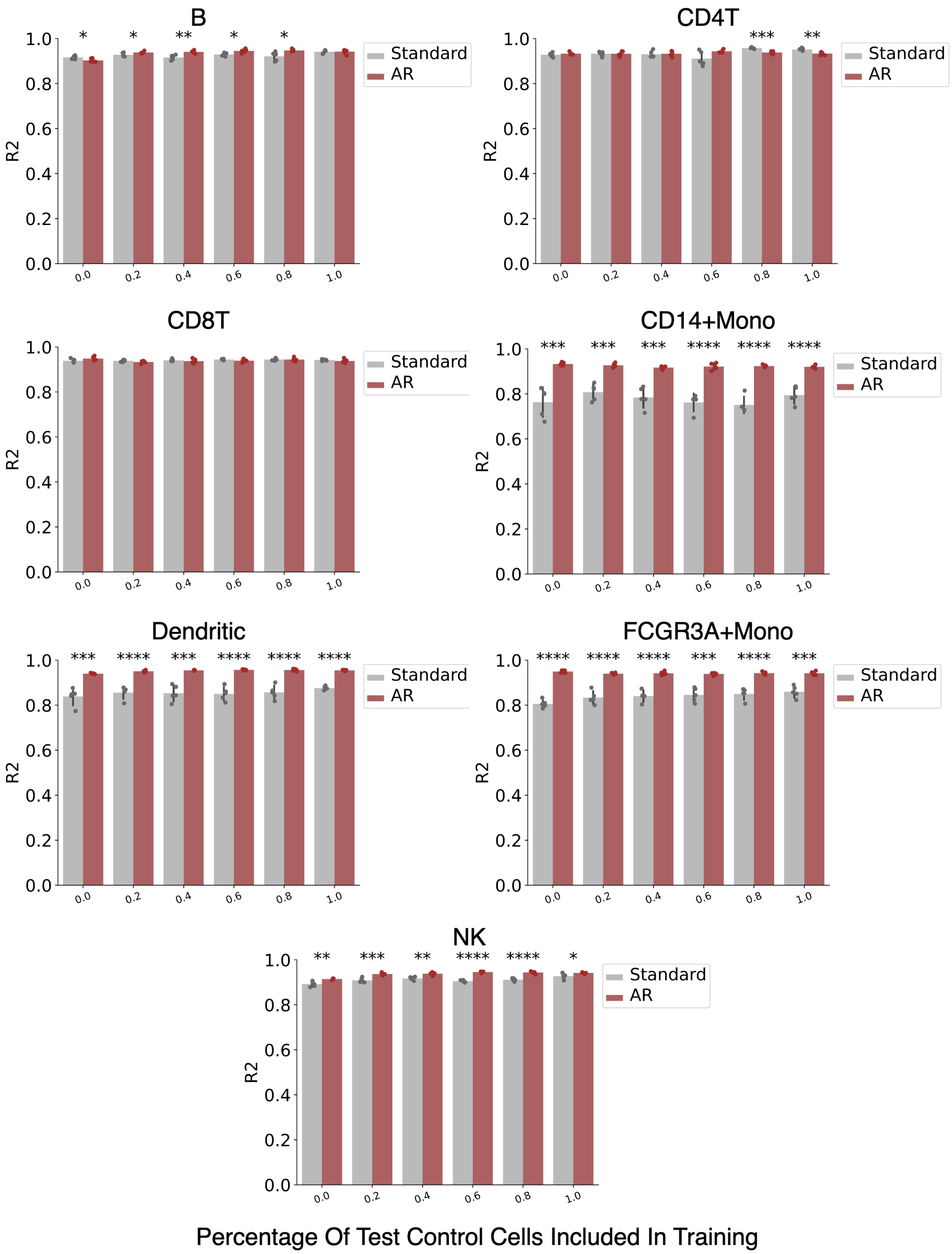

Figure S6. Adaptive resampling enables generalizable perturbation response prediction on underrepresented immune cell populations. Square of the Pearson correlation coefficient (R^2) between ground-truth and predicted perturbed gene expression in rare sample generalization scenarios on the PBMC dataset for AR versus standard models. Individual plots show results where we trained models while incrementally introducing varying proportions of control cells from the held-out group into the training corpus—specifically, 0%, 20%, 40%, 60%, 80%, and 100%. Results are based on five different random seeds, with error bars representing the standard deviations across replicates. Two-sided t-tests were performed in all experiments to compare the means of metrics, with $^{*}P < 0.05$, $^{**}P < 0.01$, $^{***}P < 0.001$, and $^{****}P < 0.0001$.

bioRxiv preprint doi: https://doi.org/10.1101/2025.11.04.686583; this version posted November 5, 2025. The copyright holder for this preprint (which was not certified by peer review) is the author/funder, who has granted bioRxiv a license to display the preprint in perpetuity. It is made available under aCC-BY 4.0 International license.

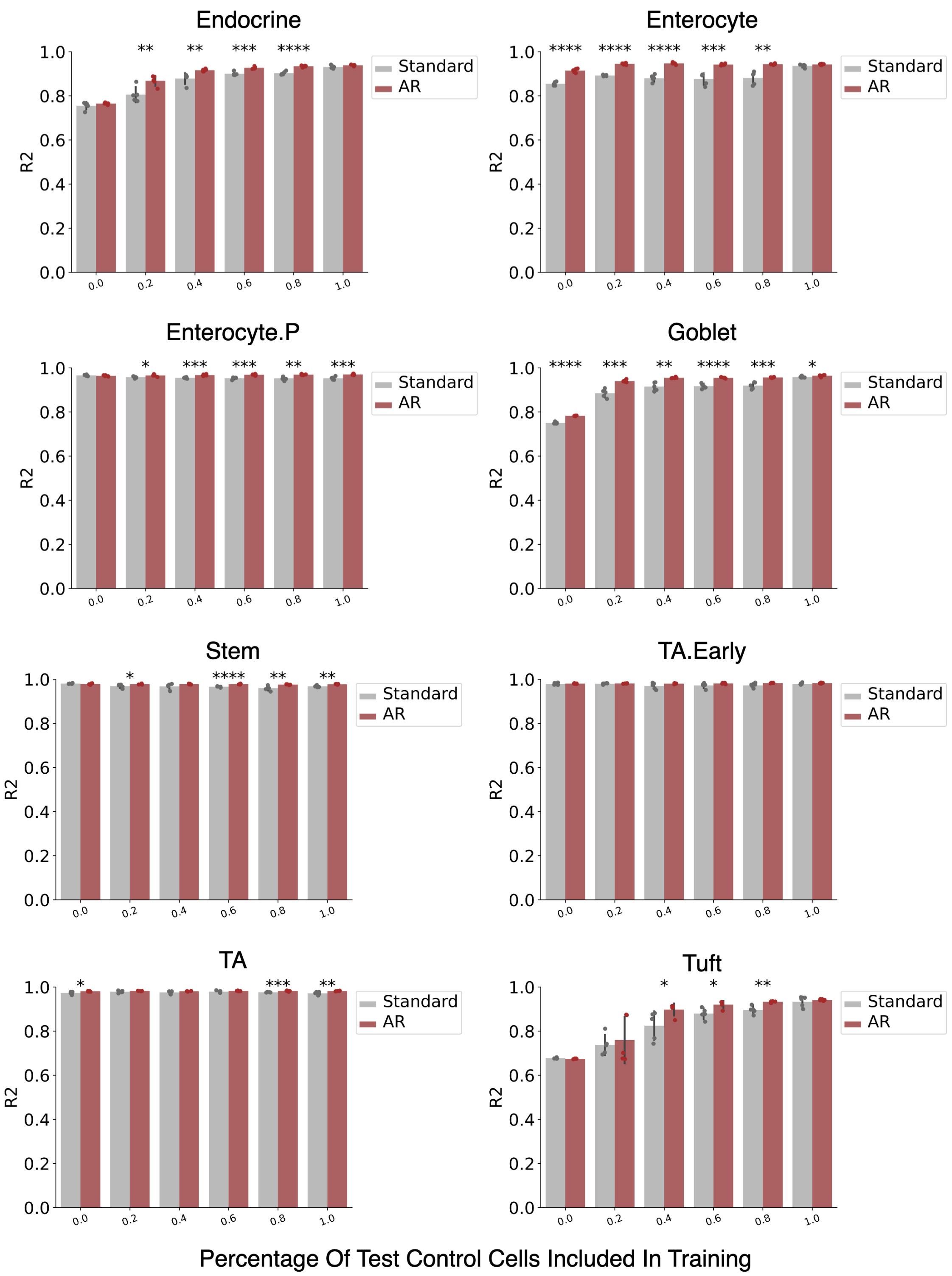

Figure S7. Adaptive resampling enables generalizable perturbation response prediction of parasitic infection in underrepresented intestinal epithelium cells. Square of the Pearson correlation coefficient (R^2) between ground-truth and predicted perturbed gene expression in rare sample generalization scenarios on the H. poly dataset for AR versus standard models. Individual plots show results where we trained models while incrementally introducing varying proportions of control cells from the held-out group into the training corpus—specifically, 0%, 20%, 40%, 60%, 80%, and 100%. Results are based on five different random seeds, with error bars representing the standard deviations across replicates. Two-sided t-tests were performed in all experiments to compare the means of metrics, with $^*P < 0.05$, $^{**}P < 0.01$, $^{***}P < 0.001$, and $^{****}P < 0.0001$.
